# The Effect of a Breakfast Rich in Slowly Digestible Starch on Glucose Metabolism: A Statistical Meta-Analysis of Randomized Controlled Trials

**DOI:** 10.3390/nu9040318

**Published:** 2017-03-23

**Authors:** Sophie Vinoy, Alexandra Meynier, Aurélie Goux, Nathalie Jourdan-Salloum, Sylvie Normand, Rémi Rabasa-Lhoret, Olivier Brack, Julie-Anne Nazare, François Péronnet, Martine Laville

**Affiliations:** 1 Mondelēz International R&D, 6 rue René Razel, 91400 Saclay, France; alexandra.meynier@mdlz.com (A.M.); aurelie.goux@mdlz.com (A.G.); 2 BIOptimize, rue Aristide Briant, 51350 Cormontreuil, France; n.jourdan@bioptimize.com; 3 Centre de Recherche en Nutrition Humaine (CRNH) Rhône-Alpes, Centre Européen Nutrition pour la santé (CENS), Centre Hospitalier Lyon Sud, Pierre Bénite and INSERM, INRA, Université Lyon 1, Hospices Civils de Lyon, 69002 Lyon, France; sylvie.normand@chu-lyon.fr (S.N.); julie-anne.nazare@cens-nutrition.com (J.-A.N.); martine.laville@chu-lyon.fr (M.L.); 4 Institut de Recherches Cliniques de Montréal, and Département de Nutrition, Faculté de Médecine, Université de Montréal, Montreal, QC H3T 1J4, Canada; Remi.Rabasa-Lhoret@ircm.qc.ca; 5 Statistique Industrielle KHI2 Consulting (KSIC), 60110 Esches, France; olivier.brack@wanadoo.fr; 6 Département de kinésiologie, Université de Montréal, Montreal, QC H3T 1J4, Canada; fperonnet@gmail.com

**Keywords:** slowly digestible starch (SDS), glycemic index (GI), glycemic response, breakfast, starch digestibility, insulin response, exogenous glucose appearance rate (RaE)

## Abstract

Starch digestibility may have an effect on the postprandial blood glucose profile. The aim of this meta-analysis was to analyze the relationship between Slowly Digestible Starch (SDS) levels and plasma glucose appearance and disappearance rates, as well as other parameters of glucose metabolism, after healthy subjects consumed cereal products that differed in SDS content. Three randomized controlled clinical trials that included a total of 79 subjects were identified. Using binary classification for the variables (high versus low levels, more than 12 g of SDS per portion, and less than 1 g of SDS per portion, respectively), we found that there was a 15-fold higher chance of having a low rate of appearance of exogenous glucose (RaE) after consumption of a high-SDS product. A high SDS content was also associated with a 12-fold and 4-fold higher chance of having a low rate of disappearance of exogenous glucose (RdE) and rate of disappearance of total plasma glucose (RdT), respectively. The RaE kinetics were further analyzed by modeling the contribution of SDS content to the different phases of the RaE response. We show that the higher the SDS content per portion of cereal product, the higher its contribution to the incremental area under the curve (iAUC) of the RaE response after 165 min. Using the association rule technique, we found that glycemic iAUC and insulinemic iAUC values vary in the same direction. In conclusion, this meta-analysis confirms the effect of the SDS level in cereal products on the metabolic response, and shows for the first time that the degree to which SDS affects the RaE response differs depending on the SDS content of the food product, as well as the phase of the postprandial period.

## 1. Introduction

Elevated postprandial glycemia may be associated with a greater risk of developing chronic metabolic diseases such as obesity, type 2 diabetes mellitus, and cardiovascular disease [1]. Carbohydrates (CHOs) are the main dietary components that affect glycemia. In 1981, Jenkins et al. introduced the concept of the glycemic index (GI), which is used to rank CHO-containing food products according to their glycemic response [2]. Several clinical trials and prospective cohort studies have shown that a low-GI diet is associated with improved glycemic control and insulin sensitivity [3,4,5,6,7]. The inverse is also true, as a meta-analysis of 37 prospective observational studies concluded that high-GI diets may increase the risk of chronic lifestyle-related diseases [8]. Due to their effect on the management of postprandial glycemic profiles, the consumption of low-GI foods has been proposed as a potential tool for improving the management of diabetes mellitus and decreasing the risk of cardiovascular disease [9,10,11,12].

The absorption kinetics of dietary CHO-derived glucose provide information that is complementary to postprandial changes in circulating plasma glucose and insulin concentrations. Although the moderate postprandial glycemic response induced by low-GI products may indicate a slower rate of appearance of ingested glucose in the systemic circulation, as well as slow tissue uptake [13], this response may also result from the rapid appearance of ingested CHOs and rapid tissue uptake. In the latter case, insulin secretion is increased, leading to a decrease in the glycemic response [14,15]. It is thus important to identify the mechanisms related to CHO absorption, to better understand the metabolic profile resulting from the difference between incoming and outgoing glucose flow rates [16]. In order to study the kinetics of absorption of CHO-rich foods, the double isotope labeling method is often used to measure the RaE in the plasma after consumption of the test food [13,17,18,19,20,21].

Starch is one of the most important glycemic CHOs present in cereal products. Starch structure, nutritional composition, and food processing methods applied to cereal products influence the rate of starch digestion [22,23,24]. Numerous studies comparing the physiological effects of starch-based products have shown a correlation between the in vitro digestibility of starch and the postprandial plasma glucose and insulin responses [25,26,27,28].

The aim of this meta-analysis was to compare the kinetics of RaE and metabolic responses after the consumption of cereal products that differed in their CHO digestibility, as represented by their content of slowly digestible starch (SDS). Complementary statistical analyses were performed to better understand the impact of SDS at different time points. The main outcomes were the relationship between the SDS content of ingested test products and 1/the RaE level, including its contribution over time to the iAUC(RaE), and 2/the postprandial glycemic response. Additional links between SDS and parameters related to the kinetics of glucose absorption (the rate of disappearance of exogenous glucose (RdE), the rate of appearance of total glucose (RaT), the rate of disappearance of total glucose (RdT), and insulinemia) were explored to better understand the mechanisms of action. In addition, the link between glycemia and insulinemia was investigated to obtain a more detailed understanding of these relationships.

## 2. Experimental Methods

### 2.1. Selection Criteria for Clinical Studies

Studies were selected if they were randomized clinical trials in humans that compared the effect of consuming low or high-SDS cereal products, as measured by the method developed by Englyst et al. [28], on the metabolic fate of exogenous glucose released from cereal products, evaluated using the double isotope labeling method.

**Figure 1:**
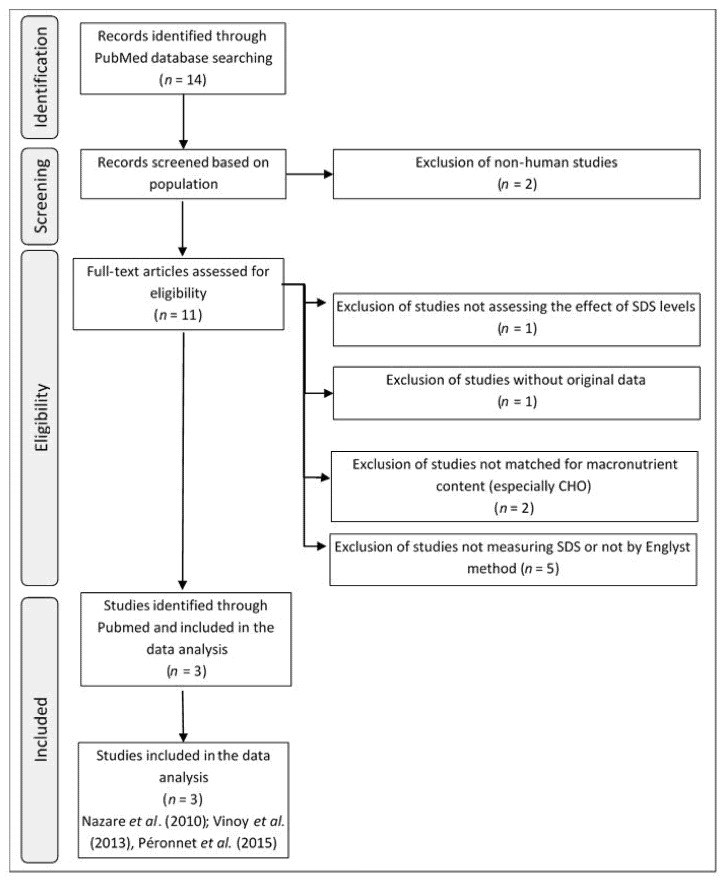
Flow chart of the different phases of the systematic review. CHO, Carbohydrates; SDS, Slowly Digestible Starch. The three selected papers were Nazare et al., Vinoy et al., and Péronnet et al. [29,30,31].

We planned to include three studies previously performed by our group in the meta-analysis [29,30,31]. A literature search was then performed to identify additional studies that had the same inclusion criteria. The selection focused on the effect of the consumption of cereal products with a known SDS content on the RaE in plasma and on the postprandial glycemic response. This literature search was performed using PubMed [32] according to the Preferred Reporting Items for Systematic Reviews and Meta-Analyses (PRISMA) statement [33]. The search strategy comprised three searches using the Boolean operator “and” that included all possible derivatives of the keywords (British English or American English terms) and searched the full text of articles, with no restrictions regarding the article type, language, text availability, or publication date (see supplemental data). Studies were excluded if they tested meals with unadjusted macronutrient contents. The search, which was performed on 29 November 2016, retrieved 13 articles. The flow chart presented in Figure 1 shows the different phases of selection of the relevant publications. After applying inclusion and exclusion criteria, three publications were selected. Only three studies corresponded to the selection criteria: Nazare et al. (2010), Vinoy et al. (2013), and Péronnet et al. (2015) [29,30,31].

### 2.2. Characteristics of the Relevant Studies

The three identified studies were randomized controlled clinical trials that analyzed the metabolic responses of healthy subjects after the consumption of a breakfast that included cereal products with either a high (biscuits) or a low (extruded cereals) SDS content. The studies followed the kinetics of both RaE and RdE in the blood using the double isotope labeling method, as well as the glycemic and insulinemic postprandial responses. The characteristics of the studies are shown in Table 1a. One was a parallel study [29], and the other two were cross-over studies.

### 2.3. Included Subjects

Sixty-six subjects (48% of whom were male) and 79 subjects (57% of whom were male) were included in the isotope and metabolic response studies, respectively (Table 1). All of the studies were performed in normo-glycemic healthy subjects with a normal body mass index (BMI), except for one study in which the subjects were overweight [29]. The overweight subjects were slightly older than the normal weight subjects.

### 2.4. Test Breakfasts

The breakfasts were consumed over a maximum period of 15 min after an overnight fast. The tested cereal products were consumed with a dairy product (skimmed, semi-skimmed, or full-fat milk) and with decaffeinated tea or coffee without sugar (or with intense sweetener) (Table 2). The breakfasts were designed to ensure that the main differences between the two meals resulted from the SDS content of the cereal products tested (0.1 to 0.9 g and 12 to 21 g SDS/portion for the low-SDS and high-SDS products, respectively). The raw ingredients used to make the cereal products in each study were the same, and the starch came from white wheat flour in both the high- and low-SDS products. The SDS contents were a result of the different manufacturing processes used to produce the cereal products. The hydrothermic parameters used to process starchy foods (temperature, moisture content, cooking time, and pressure) dramatically modify the degree of starch gelatinization. In the process used to make the high-SDS biscuits, the starch from the wheat flour is preserved from gelatinization by controlling the cooking process (moisture in the dough and temperature). In contrast, the process used to manufacture the low-SDS extruded cereals promotes a high degree of starch gelatinization, which leads to a very low SDS content (mainly due to high pressure) [16]. The other macronutrient contents were similar between the two breakfasts tested in each study. The CHO content of the breakfasts was mainly derived from the cereal product (about 80% of the total breakfast CHO content). Taking all of the studies together, the breakfasts consumed in all of the studies provided comparable amounts of energy (19%–22% of an average recommended daily calorie intake of 2000 kcal) and similar levels of CHO, protein, and fat (57%–62%, 10%–12%, and 26%–32% of the energy provided by the whole breakfast, respectively) (Table 2).

### 2.5. Experimental Conditions for Metabolic Responses and Glucose Kinetics

Blood samples were taken at baseline (before eating breakfast) and then regularly during the postprandial period (9 to 17 samples), as described in Vinoy et al. (2013) [30]. Glucose and insulin concentrations were measured for each blood sample. The glycemic responses were followed for 270 min after the subjects began to consume breakfast.

RaT and RdT were calculated from plasma D-(6,6-^2^H_2_) glucose enrichment, and RaE and RdE were determined from plasma (^13^C)-glucose enrichment [29,30,31], as described previously [34].

When the design of the studies was taken into account (parallel or cross-over, and the number of test products per subject), there were 126 and 152 data points available from the isotope and metabolic response experiments, respectively.

### 2.6. Statistical Analyses

The study subjects were compared to determine whether the study populations had similar glycemic responses, by testing the iAUC (0–120 min) for glycemia using a Welch’s ANalysis Of VAriance (ANOVA) test. The distribution of the iAUCs for glycemia was comparable in the three studies. 

Complementary statistical methods were employed to detect relationships between SDS and glucose kinetics and metabolism parameters. For the first two methods, the continuous measures required dichotomization. In order to take full advantage of continuous outcome data, a partial least squares (PLS) regression method was also used to detect relationships between descriptive and target variables.

The iAUC values for glycemia, insulinemia, RaT, and RdT represent the incremental area under the curve, ignoring the area beneath the fasting concentration, and were calculated using the trapezoidal method. The same mathematical principle was used to determine the iAUC for RaE and RdE, except that their baseline values were null. The iAUCs were calculated from T0 to the end of postprandial phase.

### 2.7. Binary Categorization and Meta-Analysis

The studied variables (SDS and iAUC of glycemic, insulinemic, RaE, RdE, RaT, and RdT responses) were categorized according to a binary classification scheme, and were considered to be qualitative variables for the purposes of the meta-analysis. The key variables to assess (related to RaE and RdE in the blood) in the two studied groups (extruded cereals and biscuits: low- and high-SDS, respectively) were categorized. A median split procedure was used to convert the continuous variables into categorical variables. This method is not affected by outliers, and enables the creation of equally sized groups. The statistical analyses were performed using MIX 2.0 Pro (Professional software for meta-analysis in Excel. Version 2.0.1.4. BiostatXL, 2011), a professional software program for performing meta-analyses. Significance tests were performed to control the risk of being misled by chance. The degree of heterogeneity in the meta-analysis was assessed using the I(2) index. As the I(2) value was 0, the studies were considered to be homogenous and to vary in the same way. A fixed effect model of SDS on the glycemic response was used to estimate the summary effect. Confidence intervals were calculated by odds ratios (OR) to assess the magnitude of the variable dependencies [35]. A z-test for association was performed to determine the statistical significance of the overall effect. The z statistic for a two-tailed test was 1.96. A statistical test greater than 1.96 or less than −1.96 would result in rejection of the null hypothesis.

### 2.8. Modeling the Relationship between the SDS Content of Products and RaE Kinetics

This analysis was performed to determine the contribution of the SDS content to RaE kinetics across the postprandial period, using continuous data. The SDS contribution was calculated by dividing the iAUC value for each 30 min period by the iAUC value over the whole postprandial period for each study. Based on these data, a PLS analysis was performed using Jump 10.0 software (SAS Institute Inc., Cary, NC, USA) to determine the contribution of SDS content and time to RaE, and to describe their interaction and quadratic terms (time × SDS and time^2^). The quadratic term SDS^2^ was not retained, as its variable importance in projection (VIP) was under the set limit (i.e., lower than 0.8). A prediction profiler was built with Excel software (version 2010) (Microsoft, Santa Rosa, CA, USA) using the prediction formula obtained from the model that was developed based on the PLS analysis.

### 2.9. Association Rules

The association rules between the glycemic and insulinemic responses were evaluated using the Apriori algorithm. The Apriori algorithm has been widely used for frequent item set mining and association rule learning in databases [36]. The Apriori algorithm aims to generate the desired rules from large item sets. In our case, the association rule learning also included binary categorization of the variables, using the median of the data from each individual study and from the three studies combined. The significance of the association rules was assessed using the “lift” value, which indicates the importance of a rule. The lift value is a measure of how much the rule deviates from a model in which the rule body and rule head (left and right items) are statistically independent. A lift value greater than 1 indicates that the rule body and the rule head appear together more often than expected, indicating that the occurrence of the rule body has a positive effect on the occurrence of the rule head [37]. The association rules are satisfied only when the support value is above 20%.

## 3. Results

### 3.1. SDS Content and RaE

**Figure 2:**
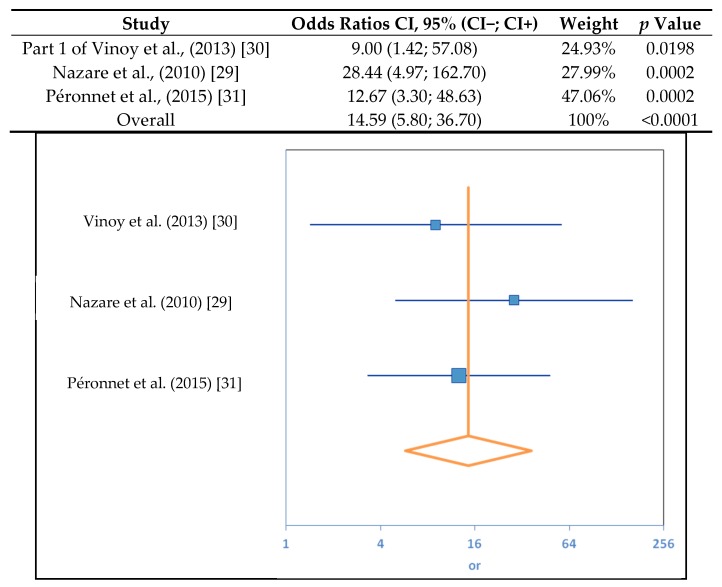
Results of the meta-analysis of the relationship between Slowly Digestible Starch (SDS) content and appearance of exogenous glucose (RaE). A median split procedure was used to convert the continuous variables into categorical variables. The degree of heterogeneity in the meta-analysis was assessed using the I(2) index. As the I(2) value was 0, the studies were considered to be homogenous and to vary in the same way. Confidence intervals were calculated by odds ratios (ORs) to assess the magnitude of the variable dependencies. A z-test for association was performed to determine the statistical significance of the overall effect. CI, confidence interval.

A meta-analysis was performed using the results from the three selected studies to determine the relationship between the SDS content of cereal products and the kinetics of glucose absorption. This analysis revealed that the RaE is significantly dependent on SDS content (*p* < 0.0001). The OR indicated that there is a 15-fold greater chance of having a low RaE after consuming a high-SDS food product compared to a low-SDS food product (Figure 2).

**Figure 3:**
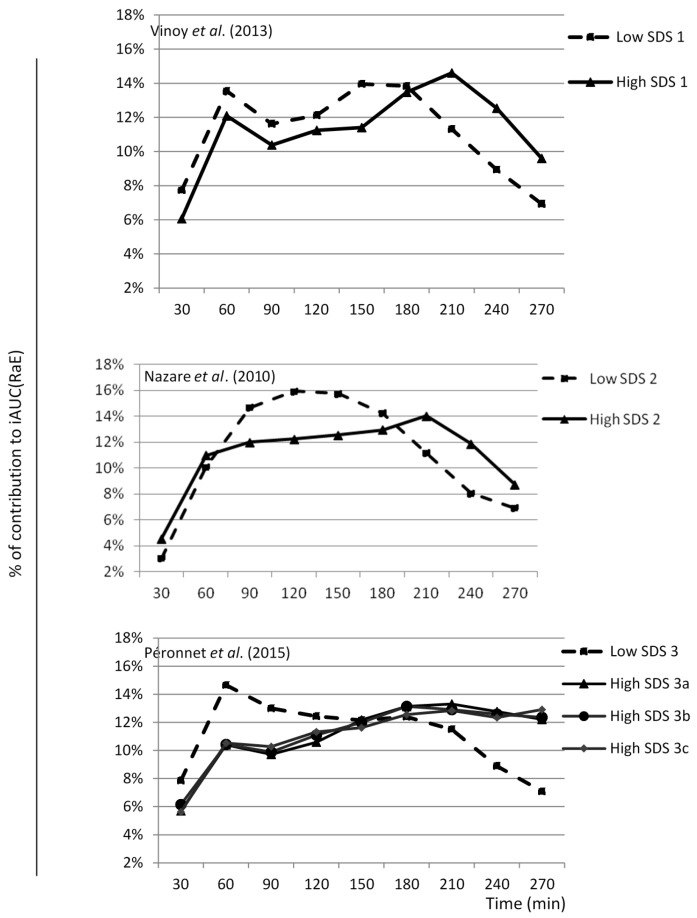
Contribution of Slowly Digestible Starch (SDS) content per portion to incremental area under the curve (iAUC) of appearance of exogenous glucose (RaE) at different time points. High-SDS products (1, 2, 3a, 3b, 3c) provided 12 to 21 g of SDS, whereas low-SDS products (1, 2, 3) provided 0 to 1 g of SDS. The SDS contribution was calculated by dividing the iAUC value for each 30 min period by the iAUC value over the whole postprandial period for each study (Vinoy et al. 2013, Nazare et al. 2010; Péronnet et al. 2015) [29,30,31]. SDS, Slowly Digestible Starch; iAUC(RaE), incremental area under the curve of the exogenous appearance rate of glucose.

**Figure 4:**
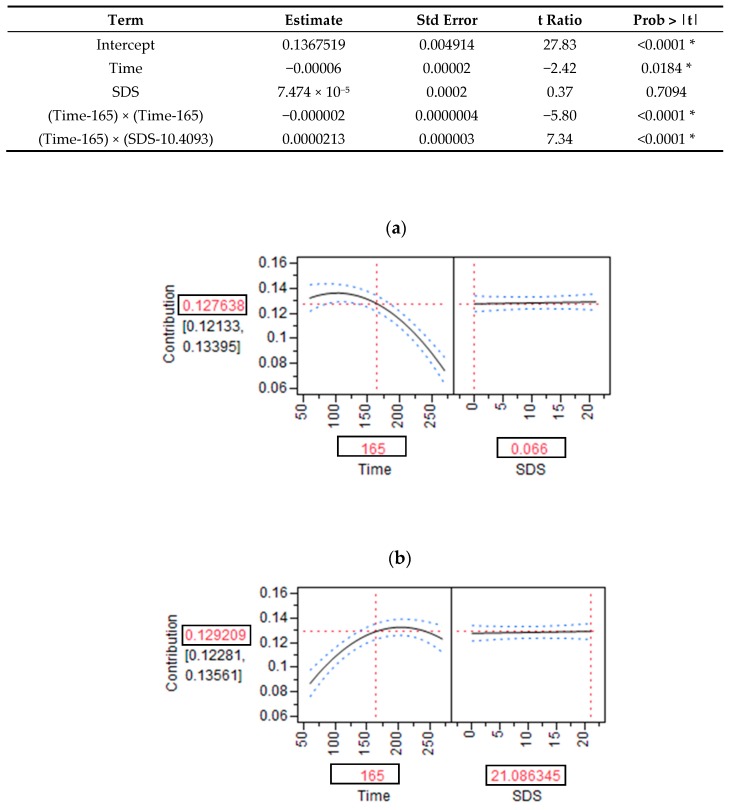
Parameter estimates and the predictive model for the contribution of Slowly Digestible Starch (SDS) content to incremental area under the curve (iAUC) of appearance of exogenous glucose (RaE), compared to the actual values from the included studies. A Partial Least Squares PLS analysis was performed to determine the contribution of SDS content and time to RaE, and to describe their interaction and quadratic terms (time × SDS and time^2^). The model explained 61% of the iAUC(RaE). A prediction profiler was built using the prediction formula obtained from the model that was developed based on the PLS analysis. The squared values indicate an example of where the dotted lines intersect the black curves: time = 165 min, contribution to the iAUC(RaE) = 13%. For an SDS content close to zero (0.07 g/portion), the greatest SDS contribution to RaE was observed between minutes 0 and 165 of the postprandial period (**a**). For high-SDS food products (21.1 g/portion), the effect of SDS content on RaE was more moderate during the first phase and more pronounced between minutes 165 and 270 min of the postprandial period (**b**). SDS, Slowly Digestible Starch; iAUC(RaE), incremental area under the curve of the exogenous appearance rate of glucose. (**a**) Profile obtained with a low SDS content (0.07 g/portion) at 165 min; (**b**) Profile obtained with a high SDS content (21.1 g/portion) at 165 min.

Next, we investigated the contribution of the SDS content to RaE kinetics at different time points. The SDS content was accounted for in the models. For each study, it appeared that the higher the SDS content of a product, the greater its contribution to the iAUC(RaE) during the late phase of the postprandial period (Figure 3). In contrast, products with lower SDS contents had a greater effect on RaE kinetics in the early phase of the postprandial period. Based on the PLS analysis, we constructed a multivariate model to describe the relationship between SDS content, sampling time, and iAUC(RaE). The model explained 61% of the iAUC(RaE), including the SDS content and time and their interaction (time x SDS), and the quadratic term time^2^. The prediction profiler illustrated this relationship, and showed that for a SDS content close to zero, the greatest SDS contribution to the RaE occurred between minutes 0 and 165 of the postprandial period (Figure 4a). Conversely, for high-SDS food products, the effect of the SDS content on RaE was more moderate during the first phase and more pronounced between 165 and 270 minutes of the postprandial period (Figure 4b).

### 3.2. SDS Content and RdE, RaT, and RdT

The association between the SDS content and RdE, RaT, and RdT was significant for all three of these kinetic parameters (Table 3). The most significant relationship observed was that between the SDS content and RdE, which is consistent with the observed association between SDS and RaE (Table 3a, *p* < 0.0001). A high SDS content was associated with a 12-fold greater chance of obtaining a low RdE and a 4-fold greater chance of obtaining a low RdT, compared to a low SDS content (Table 3a,c). There was a significant association between SDS and RaT (Table 3b, *p* = 0.0418), with a 2-fold greater chance of having a high RaT after consumption of a low-SDS product compared to a high-SDS food product. Thus, the results indicate that the SDS content is significantly associated with the kinetics of CHO absorption (confirmed by a z-test performed for each parameter), and that this association is even stronger for RaE and RdE kinetics.

### 3.3. SDS and the Glycemic and Insulinemic Responses

The analysis of the combined studies indicated that there is a significant association between the SDS content and the glycemic response, with a 5-fold greater chance of inducing a low glycemic response with a high-SDS product compared to a low-SDS product (Table 4). These results were confirmed by a z-score of 4.2 (*p* < 0.0001). A similar association was found between the SDS content and the insulin response, with a 3-fold greater chance of having a low insulinemic response in response to consumption of a high-SDS product compared to a low-SDS product; this was confirmed by a z-score of 2.8 (*p* < 0.004).

### 3.4. Association Rules between Glycemic and Insulinemic Responses

The results of the associations between the glycemic and insulinemic responses are shown in Table 5. The association rules were satisfied only when the support was greater than 20%. Four association rules were found: 73% of the cases with low glycemic iAUC have a low insulinemic iAUC, and 68% of the cases with high glycemic iAUC have a high insulinemic iAUC. Reciprocally, 73% of the cases with low insulinemic iAUC have low glycemic iAUC, and 69% of the cases with high insulinemic iAUC have high glycemic iAUC.

## 4. Discussion

This meta-analysis shows that consuming a high-SDS cereal product is associated with a 15-fold greater chance of having a low RaE in the blood and a 5-fold greater chance of having a low glycemic response, compared to consuming a low-SDS cereal product. The novelty of our meta-analysis comes from the further statistical analyses focused on the contribution of SDS levels at different time points along the postprandial RaE response curve. This is the first demonstration of the tight relationship between SDS levels in cereal products and RaE kinetics. Different levels of SDS in cereal products affect RaE kinetics differently at different time points during the postprandial period. The PLS analysis showed that the RaE response curves obtained from high-SDS and low-SDS foods crossed at 165 min after consumption of the cereal products. Products with a low SDS content had a strong impact on RaE kinetics in the first part of the postprandial period, whereas products that are high in SDS contributed less to RaE kinetics in the first part of the morning and more strongly in the second part of the morning. As both the low-SDS and the high-SDS products contained similar amounts of available CHOs, the products with a low SDS content had higher levels of rapidly digestible CHOs, which contribute strongly to the glucose kinetics during the first part of the postprandial phase. These novel results show that altering CHO digestibility alone, without modifying the macronutrient content of cereal products, is sufficient to impact the RaE.

Interestingly, our analysis showed that consumption of the high-SDS products was also strongly linked to a lower glycemic response, which was induced by the slower RaE kinetics. Previously published studies with partially comparable designs have also found a link between the slowly digestible fraction of starch and RaE [20,38,39,40]. However, the link between the slowly digestible fraction of starch and the glycemic response is not completely consistent with previously published data. Indeed, two studies from Eelderink et al. did not report any significant difference in the glycemic response, even though the products tested differed in both in vitro (SDS level) and in vivo (RaE kinetics) starch digestibility [38,39]. As mentioned previously, different methods of measuring starch digestibility can have a substantial influence on the relative estimate of glycemic potency for a given food, as the methods may not use the same cut-off point to define the slow versus rapid fractions of starch digestibility [41]. In addition, regardless of any potential methodological issues, the difference in starch digestibility between the meals used in these two studies may not have been high enough to induce significantly different glycemic responses. Indeed, the greatest difference in SDS-like content between the products used in these studies was 5 g [38,39]. In contrast, in the selected studies included in this analysis, the smallest difference in SDS content between the products was 11 g.

Finally, the positive associations found between high SDS levels and low glycemic and insulinemic responses strengthen the argument for increasing SDS levels in food products to prevent the development of metabolic diseases in healthy subjects [1,5,42,43]. High-SDS products modulate the glycemic response without exacerbating the secretion of insulin. The subjects included in the studies cited above were representative of a healthy population with normal glucose metabolism, as they all had a normal BMI or were overweight, spanned a range of ages, and were of both genders. The subjects included in the study by Nazare et al. were older than those included in the two other studies (range 20–60 years old) [29]. However, the subject comparison analysis did not reveal any significant difference between the studies in terms of the glycemic response criteria. The full analysis suggests that SDS had a similar metabolic effect even in subjects with higher BMI and age, compared to younger and normal weight subjects. Modulating the diet by including starchy foods with a high SDS content also yielded interesting results in obese insulin-resistant subjects [44]. Furthermore, in this meta-analysis, RaT, RdE, RdT, glycemic response, and insulinemic responses paralleled the RaE after the ingestion of either high- or low-SDS foods. The magnitude of all these responses was lower following ingestion of the high-SDS breakfast compared to the low-SDS breakfast. Activation of the control mechanisms responsible for restricting postprandial blood glucose excursions was, thus, precisely matched to the disturbance in plasma glucose homeostasis induced by the surge in RaE. In addition, Gastric Inhibitory Peptide (GIP) was significantly decreased in relation to the slow carbohydrate release [31].

For the purpose of this meta-analysis, we had to make some methodological choices. A systematic review was performed to select studies that compared glucose kinetics (using the double-isotope labeling method) after the consumption of cereal products with high or low SDS contents (evaluated according to the Englyst method), as well as analyzing the glycemic and insulinemic responses. The double isotope labeling method was used to measure the blood levels of endogenous and exogenous glucose originating from food, which could influence the postprandial glycemic responses. Despite an extensive literature search, the number of relevant human interventional studies fulfilling the inclusion criteria was low. Eleven studies were identified that measured glucose kinetics in blood using the double isotope labeling method. Five of these studies did not investigate the relationship between SDS levels in cereal products or the glucose kinetics and the glycemic response (Figure 1). To ensure that only the SDS level and no other components of the cereal products would influence the glucose kinetics, we excluded studies that used products in which the nutritional composition was not controlled and matched [20]. Two final publications were carefully reviewed, as they initially appeared to meet the criteria for selection [38,39]. However, to analyze the starch fractions of the food products used in these studies (one pasta and two breads made with two different types of wheat bran), the authors used an adapted version of the Englyst method [38]. As described by Woolnough et al., the method used to determine starch digestibility can have a substantial influence on the relative estimate of glycemic potency for a given food [41], which could thus bias the meta-analysis results. Therefore, we had to exclude studies using methods other than the Englyst method to characterize starch digestibility, in order to avoid any bias related to the in vitro method variability. At the end of the selection step, only three studies were identified. The weight of each study in the odds ratio determination varied according to the study design. In theory, the cross-over studies would have more impact than the parallel study [29], as all the subjects tested all the products. The weighting of the study by Péronnet et al. (2015) was also higher, as four cereal products (three of which had a high SDS content) were tested in a cross-over design, compared to two cereal products in the studies by Vinoy et al. (2013) and Nazare et al. (2010); and Péronnet et al. (2015) carried out 63 blood tests, whereas Vinoy et al. (2013) and Nazare et al. (2010) carried out 24 and 38 blood tests, respectively [29,30,31].

The three selected studies used two types of cereal products (high-SDS biscuits and low-SDS extruded cereals), in which the CHOs represented the major macronutrient (around 61% of the energy of the whole breakfast; 11% of the energy came from proteins, and 28% from fat). Additional data from a wider range of small meals that vary in SDS content, macronutrient composition, or proportion of CHOs would reinforce this body of evidence. Indeed, other studies have shown that the macronutrient content and the digestibility of CHOs can dramatically influence the metabolism of glucose derived from cereal products [17,27,45].

In the current meta-analysis, we chose to categorize the SDS values according to a binary classification scheme, because there were two clear ranges of values found in the cereal products used in the three selected studies. Indeed, the smallest difference in SDS content per meal among the selected studies was 11 g. In addition, the complementary analysis that modeled the relationship between SDS content and RaE kinetics using the continuous data yielded similar results. 

## 5. Conclusions

In conclusion, the results from this meta-analysis confirm the relationship that has already been observed in individual studies between a high SDS content in cereal food products and a low RaE in blood, as well as a moderate postprandial glycemic response. We show for the first time that the contribution of SDS to the RaE depends on the phase of the postprandial period. Starchy foods with a high SDS content contribute much more to the RaE at the end of the postprandial period than cereal products with a low SDS content. This simple modulation of cereal products to increase their SDS content reduces the challenge to plasma glucose following the meal and the associated plasma glucose and insulin excursions, and could potentially regulate additional, physiologically interesting effects, such as the prolonged elevation of gut hormone levels [43]. These phenomena may improve plasma glucose control and provide long-term health benefits [46,47].

## Figures and Tables

**Table 1:** Characteristics of the three studies included in the individual (**a**) and pooled (**b**) analyses.

**(a)**				
**Publication Reference**	**Study Design**	**Population**	**Adaptation Period**	**Number of Subjects**
Nazare et al., 2010. [29]	Randomized, parallel	Non-diabetic overweight men and pre-menopausal women (age: 20–60 years, mean 38.3; BMI: 25–30 kg/m^2^, mean 27.3 SD 1.5)	None (only day one without adaptation period)	19 (11 men and 8 women)
				19 (9 men and 10 women)
Vinoy et al., 2013. [30]	Randomized, open, cross-over (includes two parts)	Healthy subjects (age: 18–40 years, mean 24.0; BMI: 20–25 kg/m^2^, mean 22.6 SD 1.8)	3 week adaptation to tested products *	12 men (part one—isotope measurements and metabolic measurements) 13 men (part 2—metabolic measurements only)
Péronnet et al., 2015. [31]	Randomized, Open-label, cross-over	Young, lean, healthy subjects (age: 19–26 years, mean 22.0; BMI: 20.2–24.4 kg/m^2^, mean 22.0 SD 1.5)	None	16 women
**(b)**				
**Pooled Experiments**	**Number of Subjects**	**Age (Years) Mean ± SD (Min – Max) − Median**	**BMI (kg/m^2^) Mean ± SD (Min – Max) − Median**	**Weight (kg) Mean ± SD (Min – Max) − Median**
Glucose kinetics measure by isotopic method	*N* = 66 * (34 women and 32 men)	32.0 ± 10.4 (19–57) − 29	25.1 ± 3.0(20.2–30.7) − 25.9	71.6 ± 10.8 (52.6–91.2) − 70.0
Metabolic responses	*N* = 79 ** (34 women and 45 men)	30.6 ± 10.1 (19–57) – 26.0	24.8 ± 2.9 (20.2–30.7) – 25.0	72.7 ± 10.1 (52.6–98.5) − 71.9

ND, not determined; BMI, Body Mass Index; SD, Standard Deviation; Min, minimum value; Max, maximum value. * In Vinoy et al. [30], the tested breakfast products (either high-Slowly Digestible Starch (SDS) or low-SDS) were introduced into the subjects’ usual diet over a 21-day adaptation period. There was no difference in food intake between the beginning and the end of the period. The number of pooled subjects was calculated as follows: * 38 + 12 + 16 = 66 and ** 38 + 12 + 13 + 16 = 79.

**Table 2:** SDS content of the tested cereal products and composition of the whole breakfasts.

Publication Reference	Type of Cereal Products	Product Portion Size	Quantity of SDS Per Portion of Product	Foods Included in the Breakfasts Other Than Cereal Products		Composition of the Whole Breakfast			
						Energy (kcal)	CHO g (% E)	Proteins g (% E)	Fat g (% E)
Nazare et al., 2010 [29]	High-SDS product (biscuit)	80 g	21 g	Semi-skimmed milk		432	68 (62)	13 (12)	13 (26)
	Low-SDS product (extruded)	80 g	0.4 g			429	72 (62)	13 (12)	11 (26)
Vinoy et al., 2013 [30]	High-SDS product (biscuit)	70 g	12 g	Semi-skimmed milk	Tea or coffee without sugar or with intense sweetener	435	62 (57)	12 (11)	15 (32)
	Low-SDS product (extruded)	70 g	0.9 g	Whole milk		450	68 (61)	12 (11)	14 (28)
Péronnet et al., 2015 [31]	High-SDS product 1 (biscuit)	72 g	15 g	Skimmed milk	Tea or coffee without sugar or with intense sweetener	380	58 (61)	10 (10)	12 (29)
	High-SDS product 2 (biscuit)	72 g	17 g			378	58 (62)	10 (10)	12 (28)
	High-SDS product 3 (biscuit)	72 g	16 g			379	58 (62)	10 (10)	12 (28)
	Low-SDS product (extruded)	66 g	0.1 g	Skimmed milk with blend of added fats		381	58 (61)	10 (10)	12 (29)

SDS, Slowly Digestible Starch; CHO, available carbohydrates. Macronutrient content was provided in g/portion (g) and in percent of energy (% E).

**Table 3:** Results of the meta-analysis of the relationship between the SDS content and RdE (**a**), RaT (**b**), or RdT (**c**).

**(a)**				
**Study**	**OR**	**CI**	**Weight**	***p***
Part one of Vinoy et al., 2013 [30]	9.00	(1.42; 57.12)	25.22%	0.0198
Nazare et al., 2010 [29]	72.25	(9.10; 573.74)	20.06%	0.00005
Péronnet et al., 2015 [31]	7.20	(2.05; 25.24)	54.73%	0.0020
	12.09	(4.78; 30.59)	100%	0.0001
**(b)**				
**Study**	**OR**	**CI**	**Weight**	***p***
Part one of Vinoy et al., 2013 [30]	1.96	(0.39; 9.93)	22.13%	0.4164
Nazare et al., 2010 [29]	2.94	(0.79; 10.99)	33.54%	0.1088
Péronnet et al., 2015 [31]	1.89	(0.60; 5.95)	44.33%	0.2766
	2.21	[1.03; 4.74]	100%	0.0418
**(c)**				
**Study**	**OR**	**CI**	**Weight**	***p***
Part one of Vinoy et al., 2013 [30]	4.00	(0.73; 21.83)	22.24%	0.1094
Nazare et al., 2010 [29]	4.69	(1.19; 18.42)	34.23%	0.0268
Péronnet et al., 2015 [31]	3.88	(1.15; 13.05)	43.53%	0.0285
	4.17	(1.87; 9.28)	100.00%	0.0005

A median split procedure was used to convert the continuous variables into categorical variables. The degree of heterogeneity in the meta-analysis was assessed using the I(2) index. As the I(2) value was 0, the studies were considered to be homogenous and to vary in the same way. Confidence intervals were calculated by the odds ratios (OR) to assess the magnitude of the variable dependences. A z-test for association was performed to determine the statistical significance of the overall effect. SDS, Slowly Digestible Starch; RdE, disappearance rate of exogenous glucose; RaT, appearance rate of total glucose; RdT, disappearance rate of total glucose; OR, odds ratio; CI, confidence interval; z, normal standard deviation (z-distribution); p, probability; w, weight; SDS, slowly digestible starch.

**Table 4:** Results of the meta-analysis of the relationship between the SDS content and the glycemic response.

Study	OR	CI	Weight	*p*
Part one of Vinoy et al., 2013 [30]	2.00	(0.38; 10.41)	21.66%	0.4102
Nazare et al., 2010 [29]	6.43	(1.52; 27.24)	28.26%	0.0116
Part two of Vinoy et al., 2013 [30]	7.50	(1.31; 43.03)	19.31%	0.0237
Péronnet et al., 2015 [31]	6.39	(1.60; 25.48)	30.77%	0.0086
	5.13	(2.38; 11.06)	100%	0.0001

A median split procedure was used to convert the continuous variables into categorical variables. The degree of heterogeneity in the meta-analysis was assessed using the I(2) index. As the I(2) value was 0, the studies were considered to be homogenous and to vary in the same way. Confidence intervals were calculated by odds ratios (OR) to assess the magnitude of the variable dependences. A z-test for association was performed to determine the statistical significance of the overall effect. SDS, Slowly Digestible Starch; OR, odds ratio; CI, confidence interval; p, probability.

**Table 5:** Results of the evaluation of the association rules between the glycemic and insulinemic responses.

Association Rule	Support (%)	Confidence (%)	Lift
{Low glycemic iAUC}→low insulinemic iAUC	39.4	73.4	1.352
{High glycemic iAUC}→high insulinemic iAUC	31.4	67.9	1.485
{Low insulinemic iAUC}→low glycemic iAUC	39.4	72.6	1.352
{High insulinemic iAUC}→high glycemic iAUC	31.4	68.8	1.485

Incremental area under the curve (iAUC). {A}→B means the association of A with B. The Support is the percent of cases with the association rule {A}→B in the total sample. The Confidence is the percent of cases where {A}→B out of the total number of cases of {A}. For instance, 39.4% of the total subjects have low glycemic iAUC and low insulinemic iAUC, and there is 73.4% confidence of having a low insulinemic iAUC when there is a low glycemic iAUC. The significances of the association rules were assessed using the “lift” value, with a “lift” value >1.1 indicating that the association is statistically significant. iAUC, incremental area under the curve.

## References

[1] Blaak E.E., Antoine J.M., Benton D., Bjorck I., Bozzetto L., Brouns F., Diamant M., Dye L., Hulshof T., Holst J.J. (2012). Impact of postprandial glycaemia on health and prevention of disease. Obes. Rev..

[2] Jenkins D.J., Wolever T.M., Taylor R.H., Barker H., Fielden H., Baldwin J.M., Bowling A.C., Newman H.C., Jenkins A.L., Goff D.V. (1981). Glycemic index of foods: A physiological basis for carbohydrate exchange. Am. J. Clin. Nutr..

[3] Brand-Miller J., Hayne S., Petocz P., Colagiuri S. (2003). Low-glycemic index diets in the management of diabetes: A meta-analysis of randomized controlled trials. Diabetes Care.

[4] Frost G., Leeds A., Trew G., Margara R., Dornhorst A. (1998). Insulin sensitivity in women at risk of coronary heart disease and the effect of a low glycemic diet. Metab. Clin. Exp..

[5] Livesey G., Taylor R., Hulshof T., Howlett J. (2008). Glycemic response and health—A systematic review and meta-analysis: Relations between dietary glycemic properties and health outcomes. Am. J. Clin. Nutr..

[6] Livesey G., Taylor R., Livesey H., Liu S. (2013). Is there a dose-response relation of dietary glycemic load to risk of type 2 diabetes? Meta-analysis of prospective cohort studies. Am. Clin. Nutr..

[7] Rizkalla S.W., Taghrid L., Laromiguiere M., Huet D.E., Boillot J., Rigoir A., Elgrably F., Slama G. (2004). Improved plasma glucose control, whole-body glucose utilization, and lipid profile on a low-glycemic index diet in type 2 diabetic men: A randomized controlled trial. Diabetes Care.

[8] Barclay A.W., Petocz P., McMillan-Price J., Flood V.M., Prvan T., Mitchell P., Brand-Miller J.C. (2008). Glycemic index, glycemic load, and chronic disease risk—A meta-analysis of observational studies. Am. J. Clin. Nutr..

[9] Augustin L.S., Franceschi S., Jenkins D.J.A., Kendall C.W.C., La Vecchia C. (2002). Glycemic index in chronic disease: A review. Eur. J. Clin. Nutr..

[10] Jenkins D.J.A., Kendall C.W.C., Augustin L.S.A., Franceschi S., Hamidi M., Marchie A., Jenkins A.L., Axelsen M. (2002). Glycemic index: Overview of implications in health and disease. Am. J. Clin. Nutr..

[11] Salmeron J., Ascherio A., Rimm E.B., Colditz G.A., Spiegelman D., Jenkins D.J., Stampfer M.J., Wing A.L., Willett W.C. (1997). Dietary fiber, glycemic load, and risk of NIDDM in men. Diabetes Care.

[12] Salmeron J., Manson J.E., Stampfer M.J., Colditz G.A., Wing A.L., Willett W.C. (1997). Dietary fiber, glycemic load, and risk of non-insulin-dependent diabetes mellitus in women. JAMA.

[13] Normand S., Khalfallah Y., Louche-Pelissier C., Pachiaudi C., Antoine J.M., Blanc S., Desage M., Riou J.P., Laville M. (2001). Influence of dietary fat on postprandial glucose metabolism (exogenous and endogenous) using intrinsically (13)C-enriched durum wheat. Br. J. Nutr..

[14] Jenkins D.J., Ghafari H., Wolever T.M., Taylor R.H., Jenkins A.L., Barker H.M., Fielden H., Bowling A.C. (1982). Relationship between rate of digestion of foods and post-prandial glycaemia. Diabetologia.

[15] Schenk S., Davidson C.J., Zderic T.W., Byerley L.O., Coyle E.F. (2003). Different glycemic indexes of breakfast cereals are not due to glucose entry into blood but to glucose removal by tissue. Am. J. Clin. Nutr..

[16] Vinoy S., Lesdéma A., Cesbron-Lavau G., Goux A., Meynier A. (2016). Creating Food Products with a Lower Glycemic Index.

[17] Meynier A., Goux A.L., Atkinson F., Brack O., Vinoy S. (2015). Postprandial glycaemic response: How is it influenced by characteristics of cereal products?. Br. J. Nutr..

[18] Korach-André M., Roth H., Barnoud D., Péan M., Péronnet F., Leverve X. (2004). Glucose appearance in the peripheral circulation and liver glucose output in men after a large 13C starch meal. Am. J. Clin. Nutr..

[19] Livesey G., Wilson P.D.G., Dainty J.R., Brown J.C., Faulks R.M., Roe M.A., Newman T.A., Eagles J., Mellon F.A., Greenwood R.H. (1998). Simultaneous time-varying systemic appearance of oral and hepatic glucose in adults monitored with stable isotopes. Am. J. Physiol..

[20] Wachters-Hagedoorn R.E., Priebe M.G., Heimweg J.A.J., Heiner A.M., Englyst K.N., Holst J.J., Stellaard F., Vonk R.J. (2006). The rate of intestinal glucose absorption is correlated with plasma glucose-dependent insulinotropic polypeptide concentrations in healthy men. J. Nutr..

[21] Robertson M.D., Livesey G., Mathers J.C. (2002). Quantitative kinetics of glucose appearance and disposal following a ^13^C-labelled starch-rich meal: Comparison of male and female subjects. Br. J. Nutr..

[22] Granfeldt Y., Hagander B., Bjorck I. (1995). Metabolic responses to starch in oat and wheat products. On the importance of food structure, incomplete gelatinization or presence of viscous dietary fibre. Eur. J. Clin. Nutr..

[23] Lehmann U., Robin F. (2007). Slowly digestible starch—Its structure and health implications: A review. Trends Food Sci. Technol..

[24] Liljeberg H., Granfeldt Y., Bjorck I. (1992). Metabolic responses to starch in bread containing intact kernels versus milled flour. Eur. J. Clin. Nutr..

[25] Holm J., Bjoerck I. (1992). Bioavailability of starch in various wheat-based bread products: Evaluation of metabolic responses in healthy subjects and rate and extent of in vitro starch digestion. Am. J. Clin. Nutr..

[26] Heaton K.W., Marcus S.N., Emmett P.M., Bolton C.H. (1988). Particle size of wheat, maize, and oat test meals: Effects on plasma glucose and insulin responses and on the rate of starch digestion in vitro. Am. J. Clin. Nutr..

[27] Englyst K.N., Vinoy S., Englyst H.N., Lang V. (2003). Glycaemic index of cereal products explained by their content of rapidly and slowly available glucose. Br. J. Nutr..

[28] Englyst H.N., Veenstra J., Hudson G.J. (1996). Measurement of rapidly available glucose (RAG) in plant foods: A potential in vitro predictor of the glycaemic response. Br. J. Nutr..

[29] Nazare J.A., De Rougemont A., Normand S., Sauvinet V., Sothier M., Vinoy S., Désage M., Laville M. (2010). Effect of postprandial modulation of glucose availability: Short- and long-term analysis. Br. J. Nutr..

[30] Vinoy S., Normand S., Meynier A., Sothier M., Louche-Pelissier C., Peyrat J., Maitrepierre C., Nazare J.A., Brand-Miller J., Laville M. (2013). Cereal processing influences postprandial glucose metabolism as well as the GI effect. J. Am. Col. Nutr..

[31] Péronnet F., Meynier A., Sauvinet V., Normand S., Bourdon E., Mignault D., St-Pierre D.H., Laville M., Rabasa-Lhoret R., Vinoy S. (2015). Plasma glucose kinetics and response of insulin and GIP following a cereal breakfast in female subjects: Effect of starch digestibility. Eur. J. Clin. Nutr..

[32] PubMed. https://www.ncbi.nlm.nih.gov/pubmed/.

[33] Moher D., Liberati A., Tetzlaff J., Altman D.G. (2009). Preferred reporting items for systematic reviews and meta-analyses: The PRISMA statement. PLoS Med..

[34] Tissot S., Normand S., Guilluy R., Pachiaudi C., Beylot M., Laville M., Cohen R., Mornex R., Riou J.P. (1990). Use of a new gas chromatograph isotope ratio mass spectrometer to trace exogenous ^13^C labelled glucose at a very low level of enrichment in man. Diabetologia.

[35] LaMorte W.W. (2013). Case-Control Studies.

[36] Borgelt C. (2012). Frequent item set mining. WIREs Data Min. Knowl. Discov..

[37] Tufféry Stéphane (2012). Data Mining et Statistique Décisionnelle—Intelligence Des Données.

[38] Eelderink C., Moerdijk-Poortvliet T.C.W., Wang H., Schepers M., Preston T., Boer T., Vonk R.J., Schierbeek H., Priebe M.G. (2012). The glycemic response does not reflect the in vivo starch digestibility of fiber-rich wheat products in healthy men. J. Nutr..

[39] Eelderink C., Schepers M., Preston T., Vonk R.J., Oudhuis L., Priebe M.G. (2012). Slowly and rapidly digestible starchy foods can elicit a similar glycemic response because of differential tissue glucose uptake in healthy men. Am. J. Clin. Nutr..

[40] Priebe M.G., Wachters-Hagedoorn R.E., Heimweg J.A.J., Small A., Preston T., Elzinga H., Stellaard F., Vonk R.J. (2008). An explorative study of in vivo digestive starch characteristics and postprandial glucose kinetics of wholemeal wheat bread. Eur. J. Nutr..

[41] Woolnough J.W., Monro J.A., Brennan C.S., Bird A.R. (2008). Simulating human carbohydrate digestion in vitro: A review of methods and the need for standardisation. Int. J. Food Sci. Technol..

[42] Aller E.E.J.G., Abete I., Astrup A., Alfredo-Martinez J., Baak M.A. (2011). Starches, sugars and obesity. Nutrients.

[43] Vinoy S., Laville M., Feskens E.J.M. (2016). Slow-Release Carbohydrates: Growing Evidence on Metabolic Responses and Public Health Interest. Food Nutr. Res..

[44] Harbis A., Perdreau S., Vincent-Baudry S., Charbonnier M., Bernard M.C., Raccah D., Senft M., Lorec A.M., Defoort C., Portugal H. (2004). Glycemic and insulinemic meal responses modulate postprandial hepatic and intestinal lipoprotein accumulation in obese, insulin-resistant subjects. Am. J. Clin. Nutr..

[45] Garsetti M., Vinoy S., Lang V., Holt S., Loyer S., Brand-Miller J.C. (2005). The glycemic and insulinemic index of plain sweet biscuits: Relationships to in vitro starch digestibility. J. Am. Coll. Nutr..

[46] Ceriello A., Colagiuri S., Gerich J., Tuomilehto J. (2014). Guideline for management of postmeal glucose in diabetes. Diabetes Res. Clin. Pract..

[47] NaAN E.P. (2012). Guidance on the scientific requirements for health claims related to appetite ratings, weight management, and blood glucose concentrations. EFSA J..

